# Drug Eruptions

**Published:** 1887-08

**Authors:** 


DRUG ERUPTIONS. A Clinical Study of the Irritant Effects of Drugs upon the Skin. By Prince A. Morrow, A. M., M. D., Clinical Professor of Venereal Diseases; Consulting Surgeon to the Bellevue Out-door Department, etc., etc. Octavo, 206 pages, one lithographed plate. Extra muslin. Price, $1.75. New York: William Wood & Co.

This fills a niche in the world of medical books after a quite acceptable fashion. The eruptions which result from drugs often simulate so exactly the exanthemata of syphilis, and can be so easily remedied, that every practitioner should possess at least some knowledge about them. The author wisely does not attempt any artificial division of the lesions discussed. The arrangement is alphabetical, and under each drug is given careful tests by which to recognize its presence in the urine. The bibliography shows that this subject has received but little attention till within the last ten or twelve years. Here we find collected together all the material which has accumulated in this line, and also much that has not before been published. The book enters a field in which it is without a rival. H. H.

